# Aspirin Responsiveness and Early Saphenous Vein Graft Occlusion After Coronary Artery Bypass Grafting: A CT Coronary Angiography Study

**DOI:** 10.3390/medicina62020367

**Published:** 2026-02-12

**Authors:** Petar Milacic, Zoran Tabakovic, Milica Ivanovic, Ivana Petrovic, Milan Milojevic, Jelena Rakocevic, Ivan Stojanovic, Slobodan Micovic, Igor Zivkovic

**Affiliations:** 1 Dedinje Cardiovascular Institute, 11000 Belgrade, Serbia; petar_milacic@yahoo.com (P.M.); zokitaba.ikvb@ikvbd.com (Z.T.); milicaivanovicks@gmail.com (M.I.); petrovicivana4@gmail.com (I.P.); ivan0stojanovic@gmail.com (I.S.);; 2 Faculty of Medicine, University of Belgrade, 11000 Belgrade, Serbia; 3 University Hospital Zurich, University of Zurich, 8006 Zurich, Switzerland; 4 Institute of Histology and Embryology “Aleksandar Dj. Kostic”, Faculty of Medicine, University of Belgrade, 11000 Belgrade, Serbia; 5 Department of Cardiac Surgery, Dedinje Cardiovascular Institute, Heroja Milana Tepica 1, 11000 Belgrade, Serbia

**Keywords:** coronary artery bypass grafting, saphenous vein graft, aspirin responsiveness, early graft occlusion, CT coronary angiography, antiplatelet therapy

## Abstract

*Background and Objectives*: Early saphenous vein graft (SVG) failure remains a clinically significant limitation of contemporary coronary artery bypass grafting (CABG). Platelet function testing has been proposed to identify patients with an attenuated aspirin effect who may be at higher thrombotic risk. Therefore, this study aimed to determine whether preoperative aspirin non-responsiveness, assessed by the platelet function assay, is associated with early graft failure after CABG, as evaluated by CT coronary angiography. *Materials and Methods*: In this prospective observational study, consecutive patients undergoing elective, first-time isolated on-pump CABG with ≥1 SVG and preoperative aspirin therapy were enrolled. Platelet function was measured preoperatively using a point-of-care assay (ASPI, aspirin reaction units [ARU]), and patients were stratified as responders (<550 ARU) or non-responders (≥550 ARU). The primary endpoint was early SVG occlusion, detected by CT angiography performed before discharge after CABG. Secondary endpoints included postoperative cardiac and renal biomarkers, myocardial infarction, stroke, rehospitalization, and 30-day mortality. *Results*: Early CT-confirmed SVG occlusion occurred in 22/170 patients (12.9%) and did not differ between responders and non-responders (20/136 [14.7%] vs. 2/34 [5.9%]; *p* = 0.21). Cardiac biomarkers were similar between the groups for 4–24 h. Thirty-day mortality (1.5%), myocardial infarction (5.9% in each group), and stroke (2.2% vs. 5.9%) were infrequent and similar between groups. Rehospitalization was more common among non-responders, driven by deep wound infection (5.9% vs. 0%; *p* = 0.040). In exploratory analysis, females had a significantly higher early graft occlusion rate than males (27.3% vs. 8.6%; *p* = 0.004). *Conclusions*: Aspirin non-responsiveness, as assessed by ASPI testing, was not associated with early CT-confirmed SVG occlusion, and these data do not support intensifying antiplatelet therapy based solely on a single preoperative platelet-function measurement. Given the absence of serial postoperative platelet function measurements, future studies should prioritize postoperative platelet reactivity and different treatment strategies during the early window of graft vulnerability.

## 1. Introduction

In contemporary CABG, the left internal mammary artery (LIMA) is the conduit of first choice, most commonly to the left anterior descending artery, whereas additional targets are most often grafted with saphenous vein grafts (SVGs), making venous conduits the predominant “second graft” option and the most frequently used supplemental grafts overall [1]. Despite widespread use, early SVG failure remains a major limitation of surgical revascularization. Graft occlusion occurring in the early postoperative period is most frequently attributed to thrombosis, impaired distal runoff, technical factors related to graft preparation or anastomotic construction, hypercoagulable states, and progression of native coronary artery disease. Consistent with this vulnerability, SVG attrition is substantial, with 3% to 14% occluding before hospital discharge, 8% to 25% failing at 1 year, and only 50% to 60% remaining patent at 10 years [2,3,4,5,6].

Platelet activation and thrombus formation play a pivotal role in early graft failure. Accordingly, antiplatelet therapy is a cornerstone of perioperative management in patients undergoing CABG to limit graft thrombosis and its clinical sequelae. Aspirin, through irreversible inhibition of platelet cyclooxygenase-1 (COX-1), effectively suppresses thromboxane A_2_-mediated platelet aggregation and has been shown to improve early graft patency and reduce adverse cardiovascular events [7]. Although clinical practice endorses routine early aspirin with a Class I recommendation, no uniform guidance is provided on the optimal perioperative timing, dose, or the need to replace aspirin monotherapy with another P2Y12 inhibitor [8,9]. Moreover, evidence comparing aspirin monotherapy with dual antiplatelet therapy (DAPT) remains inconclusive, particularly in patients undergoing on-pump CABG, creating uncertainty regarding which subgroups may derive incremental benefit and at what bleeding cost [10].

Reduced platelet inhibition during aspirin therapy, often referred to as “aspirin resistance”, represents a clinically relevant phenomenon with potential implications for graft patency. This condition reflects inadequate suppression of platelet activity despite aspirin exposure and appears to be multifactorial, involving pharmacokinetic variability, biological factors, and patient-related characteristics [11]. Previous studies have reported aspirin non-responsiveness in 8% to 45% of patients, with a higher incidence observed in the early postoperative period after cardiac surgery as a consequence of platelet reactivity [12]. Importantly, aspirin non-responsiveness has been associated with an increased risk of thrombotic complications and adverse cardiovascular outcomes [13].

Computed tomography coronary angiography (CTA) has emerged as a reliable, non-invasive imaging modality for the assessment of bypass graft patency, with diagnostic performance approaching that of conventional coronary angiography, and its role in the early detection of clinically silent graft occlusion is increasingly recognized [14]. In parallel, point-of-care platelet function testing is being used more frequently to quantify on-treatment platelet reactivity and identify patients with suboptimal aspirin-mediated platelet inhibition [15]. Hence, the aim of this study was to evaluate early postoperative SVG patency using CTA and to assess the association between aspirin responsiveness, assessed with a point-of-care platelet function assay (ASPI), and the incidence of early graft failure following CABG.

## 2. Materials and Methods

Study design and Ethical Approval

This prospective observational study was approved by the Institutional Review Board of the Dedinje Cardiovascular Institute and the Ethics Committee of the Faculty of Medicine, University of Belgrade (Approval No. 6898, 13 December 2018). Written informed consent was obtained from all participants prior to enrollment. The study was conducted in accordance with the principles of the Declaration of Helsinki.

Study population

Consecutive patients scheduled for elective, first-time isolated CABG with implantation of at least one great saphenous vein graft were considered eligible. All procedures were performed by highly experienced cardiac surgeons, each performing more than 50 CABG procedures annually.

Inclusion criteria were:

1. Isolated CABG with at least one SVG;
2. Preoperative aspirin therapy;
3. Adequate saphenous vein diameter (2–5 mm) assessed by preoperative ultrasound mapping;
4. Vein harvesting performed by an experienced surgeon (>100 vein harvests/year).

Exclusion criteria included:

1. Combined cardiac procedures;
2. Redo CABG;
3. Emergency surgery for STEMI or NSTEMI;
4. Failed percutaneous coronary intervention with hemodynamic instability;
5. Severe peripheral arterial disease;
6. Planned total arterial revascularization;
7. Previous vein stripping;
8. Dilated or varicose great saphenous vein on ultrasound mapping.

Antiplatelet Therapy and Platelet Function Testing

All patients were on chronic aspirin therapy (100 mg daily) preoperatively, which was resumed postoperatively as early as feasible, preferably within 6 h and no later than 24 h. In cases of significant postoperative bleeding, aspirin administration was delayed until chest drainage was below 100 mL/h. P2Y12 inhibitors and anticoagulant therapy were discontinued at least seven days before surgery.

Aspirin responsiveness was assessed using the VerifyNow^®^ aspirin assay. Results were reported in aspirin reaction units (ARUs), with values < 550 ARU indicating aspirin responsiveness and ≥550 ARU indicating aspirin non-responsiveness. To avoid influencing perioperative treatment decisions, assay results were kept blinded to the clinical team and were not disclosed until recruitment was completed; accordingly, no routine postoperative platelet-function testing was performed in the absence of major bleeding complications.

Surgical Technique

All CABG procedures were performed under general anesthesia using cardiopulmonary bypass and systemic heparinization. Myocardial protection is typically achieved using antegrade cold blood cardioplegia, with dosing and redosing intervals at the discretion of the operating surgeon. A single aortic cross-clamp technique was applied in all cases. According to preoperative ultrasound mapping, we used the best part of the saphenous vein. SVGs were harvested using conventional or no-touch techniques based on intraoperative judgment and were gently distended with saline after harvesting.

CT Coronary Angiography

CT coronary angiography was performed within 7 days after surgery using a Philips Brilliance CT scanner (Philips Healthcare, Amsterdam, The Netherlands) with retrospective ECG gating and 0.67 mm slice thickness. Non-ionic contrast media were administered following a standardized protocol. Graft patency was assessed using multiplanar and volume-rendered reconstructions. Radiation exposure ranged from 5 to 15 mSv.

Study Endpoints

The primary endpoint was early SVG occlusion detected by CT coronary angiography within 7 days of index CABG. Secondary endpoints included postoperative cardiac and renal biomarkers, periprocedural myocardial infarction per the 4th Universal Definition [16], cerebrovascular events per the Academic Research Consortium-2 Definitions [17], any rehospitalization, and 30-day all-cause mortality.

Statistical Considerations and Sample Size Calculation

Sample size was determined for a two-sided comparison of proportions with α = 0.05 to detect a difference in early SVG occlusion between patients with reduced aspirin-mediated platelet inhibition and patients with aspirin responsive. We assumed an overall early SVG occlusion rate of 12% and an approximate 25% prevalence of reduced aspirin responsiveness. Based on the alternative hypothesis, we anticipated early occlusion rates of 25% in patients with reduced aspirin responsiveness versus 8% in aspirin-responsive patients, corresponding to an absolute risk difference of 17% (relative risk ≈ 3), which yields the prespecified overall event rate. With 160 evaluable patients (40 vs. 120), this design provides 80% power to detect this difference. To account for ~5% attrition or non-evaluable postoperative assessment (e.g., non-diagnostic CT, death, and withdrawal), target enrollment was set at 170 patients.

Statistical Analyses

Baseline characteristics were summarized as counts (%) for categorical variables and as the mean (±standard deviation) for approximately normally distributed continuous variables or the median (interquartile range) for non-normally distributed variables. Between-group comparisons were performed using the chi-squared test (or Fisher’s exact test when expected cell counts were small) for categorical variables and Student’s *t*-test or the Wilcoxon rank-sum test, as appropriate, for continuous variables. Serial postoperative hs-troponin concentrations were evaluated across time points using pairwise Wilcoxon tests with Bonferroni adjustment for multiple comparisons. A two-sided *p*-value < 0.05 was considered statistically significant. All analyses were performed using R version 4.2.3 (R Foundation for Statistical Computing, Vienna, Austria).

## 3. Results

The study enrolled 170 patients, stratified according to the preoperative ASPI value for aspirin responders (ASPI ≤ 550; *n* = 136) and non-responders (ASPI > 550; *n* = 34). Baseline demographics, cardiovascular risk profile, symptom burden (CCS and NYHA class), ventricular function, operative risk (EuroSCORE II), and baseline hematological parameters were largely comparable between groups (Table 1). The cohort was predominantly male (78.7% vs. 79.4%), with similar age and BMI distributions. Two differences were observed: pulmonary hypertension was more frequent among non-responders (29.4% vs. 11.0%, *p* = 0.007), and preoperative clopidogrel use was substantially higher in non-responders (41.2% vs. 11.0%, *p* < 0.001). In addition, the baseline aPTT was slightly shorter in non-responders, while INR did not differ significantly. No other preoperative characteristics differed between groups.

Preoperative angiographic and echocardiographic findings are summarized in Table 2. Overall, coronary anatomy and ventricular volumes were comparable between aspirin responders and non-responders. The distribution of two- versus three-vessel disease was similar, as was the prevalence of left main artery involvement (29.4% vs. 32.4%, *p* = 0.74) and the pattern of a concomitant left main artery plus additional vessel disease (*p* = 0.49). Target-vessel involvement did not differ between groups, with similarly high rates of LAD, circumflex and RCA disease observed; the frequency of chronic total occlusion was also comparable (49.3% vs. 50.0%, *p* = 0.94). In addition, left ventricular end-systolic and end-diastolic volumes were not significantly different (*p* = 0.31 and *p* = 0.84, respectively).

There was no significant difference in total cardiopulmonary bypass and cross-clamp times or the proportion of patients achieving complete revascularization between the two groups. Other intraoperative characteristics related to myocardial protection, coagulation and transfusion management, as well as perioperative circulatory support, were also comparable (Table 3). Notably, patients with ASPI > 550 more frequently received volatile anesthesia for maintenance (88.2% vs. 69.5%, *p* = 0.026) and more often exhibited perioperative hyperglycemia > 180 mg/dL (26.5% vs. 8.6%, *p* = 0.005).

Primary and secondary outcomes

The primary outcome, early SVG occlusion, was identified in 22 of 170 patients (12.9%) and did not differ significantly between aspirin responders and non-responders (14.1% vs. 6.1%, *p* = 0.21). Of note, in exploratory analyses, women had a markedly higher rate of early graft occlusion than men (27.3% vs. 8.6%, *p* = 0.004), corresponding to approximately fourfold higher odds of early occlusion.

Postoperative myocardial injury biomarkers other than hs-cTnT (CK and CK-MB) did not differ significantly between the ASPI groups. hs-cTnT concentrations were also similar early after surgery, with no significant between-group differences at 4 h (3504.3 [15.3–43,337.0] vs. 3489.7 [399.7–58,426.5], *p* = 0.99), 12 h (2328.3 [50.6–54,358.5] vs. 2383.8 [217.2–15,741.2], *p* = 0.67), or 24 h (1328.3 [32.0–50,213.2] vs. 1101.7 [127.2–6067.3], *p* = 0.39). At 48 h, hs-cTnT was significantly higher in responders (769.7 [31.3–23,020.8] vs. 325.9 [111.0–8213.6], *p* = 0.002). Overall, hs-cTnT decreased significantly over time in both groups (post hoc pairwise Wilcoxon tests with Bonferroni correction, *p* < 0.001).

Serum creatinine changed significantly over time in both groups (post hoc pairwise Wilcoxon tests with Bonferroni correction, *p* < 0.001). Preoperatively, creatinine values were comparable between aspirin responders and non-responders (76.5 [5.0–294.3] vs. 73.9 [49.2–178.1], *p* = 0.184). Postoperatively, creatinine was modestly higher in responders, including at discharge (78.5 [34.6–2202.0] vs. 70.4 [42.1–139.5], *p* = 0.032).

Re-exploration for increased chest tube drainage was required in nine patients (5.3%), with no significant difference between aspirin responders and non-responders (6/136 [4.4%] vs. 3/34 [8.8%], *p* = 0.30). Perioperative myocardial infarction occurred in 10 patients (5.9%) and was similar between groups (8/136 [5.9%] vs. 2/34 [5.9%], *p* > 0.99). Stroke events were observed in five patients (2.9%), with a numerically higher but non-significant rate in non-responders (2/34 [5.9%] vs. 3/136 [2.2%], *p* = 0.25). Rehospitalization within 30 days was more frequent in non-responders (2/34 [8.8%] vs. 2/136 [1.5%], *p* = 0.025), most commonly due to deep wounds or sternal infection (2/34 [5.9%] vs. 0/136 [0%], *p* = 0.04).

Thirty-day mortality was low and did not differ between groups (2/136 [1.5%] vs. 0/34 [0%], *p* > 0.99). Postoperative biomarkers, aspirin administration, early clinical course, and 30-day outcomes are summarized in detail in Table 4.

## 4. Discussion

The present study provides important perspectives on the clinical relevance of reduced aspirin responsiveness for early SVG failure, as assessed by CT coronary angiography. Our findings indicate that the proportion of patients classified as having reduced aspirin responsiveness using the prespecified ASPI cut-off (>550) was 20%, which is lower than that reported in several prior CABG cohorts. Second, reduced preoperative aspirin responsiveness was not associated with a higher incidence of early SVG occlusion or major ischemic events during early follow-up. In the overall cohort, early CTA-confirmed graft occlusion occurred in 12.9% of patients, consistent with published estimates for early postoperative SVG failure, and the event rate did not differ between ASPI-defined responders and non-responders. Beyond the primary endpoint, perioperative myocardial infarction, stroke, and 30-day mortality were similarly uncommon and comparable across groups, suggesting that preoperative platelet-function status alone may be insufficient to identify patients at excess risk of clinically relevant early thrombotic events. Finally, exploratory analyses suggested female sex as a potential risk factor for early graft occlusion, with females experiencing substantially higher occlusion rates than men, underscoring that patient-level factors, such as sex, unrelated to ASPI status, may have a stronger influence on early graft patency.

The great saphenous vein remains the most frequently used conduit for coronary revascularization; however, its major limitation is the persistently high incidence of early graft failure, which has not been meaningfully reduced by several secondary prevention strategies, including aggressive lipid-lowering with PCSK9 inhibitors [18]. CT coronary angiography with contemporary multidetector scanners provides a safe and reliable, non-invasive assessment of bypass graft patency, with diagnostic performance approaching that of invasive coronary angiography [19,20], thereby enabling systematic evaluation of this clinically important adverse event and supporting targeted efforts to reduce its incidence. In our cohort, early SVG occlusion occurred in 13% of patients, consistent with published estimates for the early postoperative period and underscoring that early graft failure remains common [21]. While technical factors (e.g., conduit handling, anastomotic quality, graft configuration, and distal runoff) are important determinants of early failure [22], harvesting, handling, and manipulation of the saphenous vein can compromise endothelial integrity, thereby promoting platelet activation, adhesion, and thrombus formation. Platelet activation and thrombin generation have been identified as key pathophysiological processes underlying early saphenous vein graft failure [23,24], and these prothrombotic mechanisms may be further amplified in the setting of cardiopulmonary bypass [25]. However, baseline imbalances, particularly more frequent preoperative clopidogrel exposure and differences in aPTT in the non-responder cohort, may have introduced residual confounding and should be considered when interpreting these findings.

Irreversible inhibition of platelet cyclooxygenase-1 by aspirin is a cornerstone of secondary prevention and has been shown to reduce the risk of stroke, myocardial infarction, and vascular death in patients with chronic coronary syndromes [26]. In the CABG setting, early postoperative platelet inhibition is particularly relevant, as the highest risk of SVG thrombosis occurs in the immediate postoperative period. Accordingly, aspirin, administered as early as feasible, ideally within the first 6 h and no later than 24 h when bleeding is controlled, has been associated with improved early graft patency and fewer ischemic complications. During the first postoperative year, routine aspirin therapy appears to provide vein graft patency benefits comparable to those reported with ticagrelor monotherapy [27], while offering a long-established safety profile, more predictable pharmacodynamic effects, substantially lower costs, and worldwide availability. Preoperative aspirin use is also advocated as safe and has been associated with lower operative morbidity and mortality [8,28]. For years, variability in aspirin’s antiplatelet effect, often labeled “aspirin resistance”, has been proposed as a contributor to early graft thrombosis and has driven interest in routine platelet-function screening [29,30]. However, much of the evidence comes from heterogeneous, largely retrospective studies using different assays with limited agreement and inconsistent associations with outcomes, leaving its clinical relevance after CABG uncertain [31]. Moreover, the term “resistance” may be misleading, as attenuated aspirin effect often reflects modifiable pharmacodynamic variability in high platelet-turnover states (e.g., on-pump CABG) and may improve with optimized dosing, adherence, and avoidance of competing NSAIDs [32,33]. This study indicates that while 20% of patients showed reduced responsiveness to aspirin based on the prespecified ASPI threshold, this early preoperative finding was not linked to initial CTA-confirmed SVG occlusion or early ischemic events. Although baseline differences between ASPI groups, such as preoperative clopidogrel exposure and aPTT, could have introduced residual confounding, these imbalances would likely favor higher occlusion rates in non-responders, not lower. Therefore, the findings suggest that routinely intensifying antiplatelet therapy after a single preoperative platelet-function test may not be necessary, and dynamic perioperative and postoperative factors might be more critical in determining early graft thrombosis.

Dual antiplatelet therapy (DAPT), combining aspirin with a P2Y12 inhibitor, has been proposed to enhance platelet inhibition, reduce aspirin responsiveness, or heighten thrombotic risk. However, evidence supporting routine DAPT after elective CABG remains limited, as no randomized clinical trials have been sufficiently powered to assess individual clinical outcomes of graft failure or all-cause death [8]. An individual patient-data meta-analysis of four randomized trials, including 1316 patients and 1668 saphenous vein grafts, showed that adding ticagrelor to aspirin for 12 months reduced the risk of vein graft failure, but at the expense of a significantly increased risk of clinically important bleeding [10]. This trade-off has fueled uncertainty about the net clinical benefit of prolonged DAPT in unselected CABG populations and has prompted efforts to define shorter, safer strategies. In this context, ongoing studies are evaluating whether 3-month or 1-month ticagrelor-based DAPT regimens may be non-inferior to prolonged DAPT with respect to SVG patency, and whether subsequent de-escalation to aspirin monotherapy can reduce clinically relevant bleeding without an apparent increase in ischemic events (TOB-CABG trial, ClinicalTrials.gov NCT05380063; ODIN trial, NCT05997693) [34]. Collectively, these investigations should clarify whether any incremental graft-patency benefit of DAPT outweighs the associated bleeding risk and whether shortening DAPT duration represents a more balanced strategy in selected patients with chronic coronary syndromes following CABG. Importantly, in the acute setting, recent results from the TACSI trial showed that at 12 months, the incidence of major adverse cardiovascular events was similar between DAPT and aspirin monotherapy, whereas major bleeding was significantly higher with DAPT [35]. These findings further reinforce the role of aspirin monotherapy as a default antiplatelet strategy after CABG, not only in chronic coronary syndromes but also in patients operated on in the context of acute coronary syndromes.

Several studies have found an increased risk of death and complications in females following CABG [36]. A retrospective study using data from the Society of Thoracic Surgeons Adult Cardiac Surgery Database, which included almost 320,000 women who underwent surgery from 2011 to 2020, confirmed that women face a significantly higher risk of adverse outcomes after coronary artery bypass grafting, with little improvement over the past decade [37]. Prior research has also reported higher saphenous vein graft occlusion rates in females at 1 year, potentially related to smaller coronary artery diameter, more diffuse coronary disease, and fewer suitable graft targets [38]. This aligns with our observation that female sex was associated with a markedly higher rate of early graft occlusion, with women exhibiting approximately fourfold higher odds of early occlusion compared to men. In addition, several factors, including perioperative hemodilution, anemia, and transfusion exposure, which are often more frequent in females, may further contribute to sex-based differences in outcomes and could plausibly interact with early thrombotic and bleeding risk. These considerations highlight the need to better characterize sex-specific determinants of graft failure and optimize perioperative antithrombotic strategies. Ongoing dedicated research, including ROMA:Women (ClinicalTrials.gov Identifier: NCT04124120) [39], should help clarify mechanisms and inform more individualized approaches for females undergoing CABG.

Study limitations

The present study has several limitations inherent to its design. Platelet function was assessed using a single assay at a single preoperative time point, without serial postoperative measurements that may better capture the dynamic perioperative hemostatic milieu. The relatively small non-responder subgroup and low event counts limit statistical power, particularly to detect smaller, yet potentially clinically meaningful, between-group differences, and restrict the robustness of subgroup comparisons. No-touch SVG harvesting was limited (approx. 5%), precluding meaningful assessment of its impact on early graft patency. In addition, the absence of multivariable modeling limits causal inference and does not account for potential residual confounding, particularly given baseline imbalances in preoperative clopidogrel exposure and aPTT between responders and non-responders. Although treatment decisions were not guided by platelet-function results, the study was not placebo-controlled and, therefore, cannot fully exclude the possibility of performance bias, even if this is less likely to affect hard endpoints such as death or myocardial infarction. Follow-up was focused on early (30-day) outcomes; therefore, any longer-term clinical implications of differences in graft patency, including later graft attrition, repeat revascularization, or downstream ischemic events, may not have been captured. Finally, while CT angiography provides reliable detection of graft occlusion, the clinical significance of silent occlusions likely varies by target territory, collateralization, and completeness of revascularization.

## 5. Conclusions

Early saphenous vein graft occlusion remains a clinically important limitation of contemporary CABG; CT coronary angiography enables reliable, non-invasive detection of early graft failure. In this cohort, 20% of patients met the prespecified threshold for reduced aspirin responsiveness (ASPI ≥ 550 ARU), yet ASPI-defined non-responsiveness was not associated with early CTA-confirmed SVG occlusion or early ischemic events. These data do not support intensifying antiplatelet therapy solely on the basis of a single preoperative platelet-function measurement. Given the multifactorial nature of early graft failure and the highly dynamic hemostatic and platelet milieu after CABG, future research should evaluate whether postoperative platelet reactivity profiling and treatment strategies guided by it can improve graft patency while maintaining an acceptable bleeding risk. Until such evidence is available, aspirin monotherapy should remain the default antiplatelet strategy after CABG, while the role, duration, and patient selection for other antiplatelet strategies should be individualized and tested in adequate trials using clinically relevant endpoints.

## Figures and Tables

**Table 1:** Baseline characteristics.

Demographics		ASPI Value		
		≤550	>550	*p*-Value
Male sex		107 (78.7%)	27 (79.4%)	0.92
Age		66.5 (77.0–28.0)	65.0 (50.0–82.0)	0.79
BMI (kg/m^2^)		27.7 (19.1–41.5)	27.8 (22.2–37.8)	0.74
Smoking history, any		95 (69.9%)	20 (58.8%)	0.22
Diabetes mellitus		69 (50.7%)	22 (64.7%)	0.14
Medically treated hypertension		135 (99.3%)	34 (100%)	>0.99
Hyperlipidemia		130 (95.6%)	31 (91.2%)	0.30
Family history for CAD		91 (66.9%)	19 (55.9%)	0.23
Peripheral vascular disease		22 (16.2%)	6 (17.6%)	0.84
Cerebrovascular disease		10 (7.4%)	3 (8.8%)	0.77
Chronic obstructive pulmonary disease		11 (8.1%)	1 (2.9%)	0.29
Creatinine value		85.6 (49.7–530.6)	81.4 (52.6–245.6)	0.21
Previous IM		65 (49.2%)	20 (58.8%)	0.32
Previous PCI		31 (24.2%)	4 (12.5%)	0.15
CCS AP	0	19 (14.0%)	5 (14.7%)	0.80
	I	29 (21.3%)	10 (29.4%)	
	II	47 (34.6%)	9 (26.5%)	
	III	12 (8.8%)	2 (5.9%)	
	IV	29 (21.3%)	8 (23.5%)	
NYHA	1	9 (6.6%)	3 (8.8%)	0.89
	2	76 (55.9%)	19 (55.9%)	
	3	49 (36.0%)	11 (32.4%)	
	4	2 (1.5%)	1 (2.9%)	
LVEF	≥50%	52 (38.5%)	16 (48.5%)	0.52
	40–49%	52 (38.5%)	12 (36.4%)	
	30–39%	20 (14.8%)	2 (6.1%)	
	<30%	11 (8.1%)	3 (9.1%)	
Congestive Heart Failure		14 (10.3%)	3 (8.8%)	0.80
Pulmonary hypertension		15 (11.0%)	10 (29.4%)	0.007
Baseline clinical score, hematological parameters and medication at the hospital admission				
EuroSCORE II		1.51 (0.50–9.42)	1.28 (0.76–3.35)	0.48
Hgb		135.2 ± 14.8	134.3 ± 16.0	0.66
Hct		40.1 (28.5–49.3)	39.0 (27.1–65.2)	0.87
Er		4.56 (3.06–7.69)	4.46 (3.20–6.19)	0.44
Leu		7.1 (3.2–15.9)	6.7 (4.0–10.4)	0.28
aPTT		30.8 (21.9–96.9)	29.7 (11.2–40.9)	0.021
INR		1.11 (0.90–1.42)	1.16 (0.91–2.96)	0.076
ACE/ARB/ARNI		129 (94.9%)	31 (91.2%)	0.41
B blockers		130 (95.6%)	31 (93.9%)	0.69
Ca channel blockers		46 (34.1%)	11 (32.4%)	0.85
Nitrates		40 (29.4%)	14 (41.2%)	0.19
Statins		119 (88.1%)	32 (94.1%)	0.31
Amiodarone		8 (5.9%)	4 (11.8%)	0.23
Clopidogrel		15 (11.0%)	14 (41.2%)	<0.001

ASPI, aspirin platelet-function assay value (aspirin reaction units); BMI, body mass index; CAD, coronary artery disease; IM, myocardial infarction; PCI, percutaneous coronary intervention; CCS AP, Canadian Cardiovascular Society angina pectoris class; NYHA, New York Heart Association functional class; LVEF, left ventricular ejection fraction; EuroSCORE II, European System for Cardiac Operative Risk Evaluation II; Hgb, hemoglobin; Hct, hematocrit; Er, erythrocyte count (red blood cell count); Leu, leukocyte count (white blood cell count); aPTT, activated partial thromboplastin time; INR, international normalized ratio; ACE, angiotensin-converting enzyme; ARB, angiotensin receptor blocker; ARNI, angiotensin receptor–neprilysin inhibitor; Ca, calcium.

**Table 2:** Coronary anatomy and ventricular volumes stratified by ASPI group.

Patient Characteristics		ASPI		
		≤550	>550	*p*-Value
Extent of coronary artery disease				
Two-vessel disease		28 (20.6%)	8 (23.5%)	0.91
Three-vessel disease		105 (77.2%)	26 (76.5%)	
LM involvement		40 (29.4%)	11 (32.4%)	0.74
LM plus additional vessel disease	LM + 1VD	3 (7.5%)	0	0.49
	LM + 2VD	8 (20.0%)	4 (36.4%)	
	LM + 3VD	29 (72.5%)	7 (63.6%)	
Target-vessel involvement				
LAD		131 (96.3%)	34 (100%)	0.26
Cx		128 (94.1%)	29 (85.3%)	0.083
RCA		123 (90.4%)	32 (94.1%)	0.50
CTO, any		67 (49.3%)	17 (50.0%)	0.94
Left ventricular volumes				
LVESV, mL		36.5 (25.0–59.0)	35.0 (27.0–51.0)	0.31
LVEDV, mL		53.0 (23.0–71.0)	52.5 (47.0–68.0)	0.84

ASPI, aspirin assay value (aspirin reaction units; ARU); LM, left main coronary artery; VD, vessel disease; LAD, left anterior descending coronary artery; Cx, left circumflex coronary artery; RCA, right coronary artery; CTO, chronic total occlusion; LVESV, left ventricular end-systolic volume; LVEDV, left ventricular end-diastolic volume. Values are expressed as percentages (*n*/N), and continuous variables are presented as median (interquartile range).

**Table 3:** Intraoperative and perioperative procedural characteristics.

Characteristics	ASPI		
	≤550	>550	*p*-Value
Procedure and perfusion			
Cardiopulmonary bypass time, min	87 (27–197)	91.5 (59.0–284.0)	0.20
Cross-clamp time, min	58 (0–154)	56.5 (0–123.0)	0.90
No-touch technique of vein harvesting	7 (5.1%)	2 (5.9%)	0.89
Complete revascularization	107 (79.9%)	29 (87.9%)	0.29
Myocardial protection and temperature			
Repeated cardioplegia	26 (19.3%)	8 (23.5%)	0.58
Total cardioplegia volume, mL	1200 (600–2200)	1200 (800–2100)	0.68
Lowest Temperature, °C	33.0 (30.1–35.0)	33.0 (31.0–36.0)	0.35
Anesthesia			
Volatile anesthetics for maintenance	89 (69.5%)	30 (88.2%)	0.026
Metabolic control			
Glycemia > 180 mg/dL	11 (8.6%)	9 (26.5%)	0.005
Hyperglycemia duration, min.	95 (30–180)	105 (30–250)	0.36
Lowest Hemoglobin	89 (54–117)	91 (64–129)	0.18
Coagulation management			
ACT before CPB start	561.4 ± 69.8	532.8 ± 45.6	0.98
Heparin dose, IU	35,000 (11,500–47,500)	32,000 (25,000–40,000)	0.16
Protamine dose, mg	300 (200–450)	300 (200–400)	0.20
Final ACT	121 (98–215)	116 (105–166)	0.41
Antifibrinolytic use	133 (98.5%)	34 (100%)	>0.99
Circulatory support			
Inotropes at CPB weaning	31 (22.8%)	9 (26.5%)	0.65
Vasopressors at CPB weaning	60 (44.1%)	15 (44.1%)	>0.99
Temporary mechanical circulatory support, any	1 (0.7%)	0	>0.99
Temporary pacemaker implantation	66 (48.5%)	16 (47.1%)	0.88
Transfusion			
PRBC transfusion	21 (15.4%)	4 (11.8%)	0.59
Platelets transfusion	1 (0.7%)	0	>0.99

ASPI, aspirin platelet-function assay value (aspirin reaction units); CPB, cardiopulmonary bypass; ACT, activated clotting time; PRBC, packed red blood cells. Continuous variables are presented as median (interquartile range) unless otherwise indicated; ACT before CPB starts is presented as mean ± standard deviation. Values are expressed as percentages (n/*N*), and continuous variables are presented as median (interquartile range). ACT before CPB starts is presented as mean ± standard deviation.

**Table 4:** Postoperative biomarkers, aspirin administration, and early clinical course stratified by ASPI group.

Cardiac and Renal Biomarkers	ASPI		
	≤550	>550	*p*-Value
4 h post-op			
hs-cTnT	3504.3 (15.3–43,337.0)	3489.7 (399.7–58,426.5)	0.99
CK	380.5 (35–1728)	406 (175–1327)	0.098
CK-MB	31 (10–254)	33.5 (13–113)	0.34
Creatinine	77.9 (39.4–417.8)	73.8 (45.6–167.1)	0.11
Drainage	150 (0–600)	175 (0–500)	0.27
12 h post-op			
hs-cTnT	2328.3 (50.6–54,358.5)	2383.8 (217.2–15,741.2)	0.67
CK	419.5 (51–3037)	528.5 (122–1229)	0.12
CK-MB	28 (11–843)	30 (12.6–79.0)	0.41
Creatinine	82.8 (39.4–588.0)	74.6 (50.6–996.0)	0.21
Drainage 12 h	350 (50–1750)	350 (50–1350)	0.81
24 h post-op			
hs-cTnT	1328.3 (32.0–50,213.2)	1101.7 (127.2–6067.3)	0.39
CK	429 (19–2693)	466 (132–4549)	0.75
CK-MB	21 (9–363)	23.7 (11.0–71.3)	0.86
Creatinine	77.4 (33.7–535.0)	68.4 (42.5–217.7)	0.017
Drainage	500 (50–2200)	500 (150–1550)	0.36
48 h post-op			
hs-cTnT	769.7 (31.3–23,020.8)	325.9 (111.0–8213.6)	0.002
CK	348 (23–4892)	323 (82–1612)	0.34
CK-MB	16 (6–315)	14.3 (7.0–36.7)	0.18
Creatinine	80.3 (43.6–1098.0)	75.2 (40.8–317.3)	0.32
Drainage	700 (100–3300)	700 (150–2450)	0.70
Discharge			
hs-cTnT	91.7 (5.4–2543.8)	68.2 (12.7–2998.0)	0.074
CK	87 (4–1135)	68.5 (26–1645)	0.146
CK-MB	11 (4–128)	10 (4–31)	0.408
Creatinine	78.5 (34.6–2202.0)	70.4 (42.1–139.5)	0.032
Total drainage, mL	700 (100–3300)	750 (150–4500)	0.669
Aspirin administration and early clinical outcomes			
Aspirin within 24 h	128 (94.1%)	34 (100%)	0.15
Aspirin within 72 h	134 (98.5%)	34 (100%)	>0.99
P2Y12 inhibitors within 72 h	2 (1.83%)	1 (3.2%)	0.52
Mechanical ventilation, hours	10.0 (4.0–40.0)	10.5 (7.0–24.0)	0.086
ICU stay, hours	24 (12–169)	24 (16–144)	0.20
Lowest Hgb in the ICU	103.0 ± 14.8	102.6 ± 14.4	0.88
PRBC, total	40 (29.4%)	15 (44.1%)	0.10
Anticoagulation treatment	20 (20.8%)	6 (33.3%)	0.18
Reintervention for bleeding	6 (3.4%)	3 (8.8%)	0.30
30-day outcomes			
Graft occlusion	20 (14.7%)	2 (5.9%)	0.21
All-cause death	2 (1.5%)	0	>0.99
Myocardial infarction	8 (5.9%)	2 (5.9%)	>0.99
Stroke	3 (2.2%)	2 (5.9%)	0.25
Deep wound infection	0	2 (5.9%)	0.040
Superficial wound infection	8 (5.9%)	3 (8.8%)	0.54
Rehospitalization	2 (1.5%)	2 (8.8%)	0.025

ASPI, aspirin platelet-function assay value (aspirin reaction units); hs-cTnT, high-sensitivity cardiac troponin T; CK, creatine kinase; CK-MB, creatine kinase–MB fraction; ICU, intensive care unit; Hgb, hemoglobin; PRBC, packed red blood cells; P2Y12 inhibitor, adenosine diphosphate (ADP) P2Y12 receptor inhibitor; mL, milliliters.

## Data Availability

The original contributions presented in this study are included in the article. Further inquiries can be directed to the corresponding author.
